# Supplementary material for: Pediatrics HIV-positive status disclosure and its predictors in Ethiopia: a systematic review and meta-analysis
Source: PeerJ. 2022 Aug 23;10:e13896. doi: 10.7717/peerj.13896 (PMC9415365; doi:10.7717/peerj.13896)
Supplement: Supplemental Information 2 [file peerj-10-13896-s002.docx]

**The rationale for conducting the systematic review and meta-analysis**

HIV status disclosure is very challenging in pediatrics, this study is used to estimate the appropriate time for HIV disclosure. However, there are no studies that revealed the overall estimate of pediatric HIV-positive status disclosure in Ethiopia. Therefore, this systematic review and meta-analysis aimed to estimate the national pooled estimate of HIV-positive serostatus disclosure and its predictors among children.

**The contribution that it makes to the knowledge in light of previously published related reports, including other meta-analyses and systematic reviews.**

Despite worries about the communal and psychological impacts of disclosure to children some studies in developed countries showed that HIV positive children changed well after disclosure than children unaware of their HIV status. Studies revealed that fear of violence, desertion or blame, stigma, and discrimination are some of the barriers to disclosing children’s HIV-positive status
